# Supplementary material for: Global and regional importance of the direct dust-climate feedback
Source: Nat Commun. 2018 Jan 16;9:241. doi: 10.1038/s41467-017-02620-y (PMC5770443; doi:10.1038/s41467-017-02620-y)
Supplement: Supplementary file 1 — Supplementary Information [file 41467_2017_2620_MOESM1_ESM.pdf]

Supplementary Figures

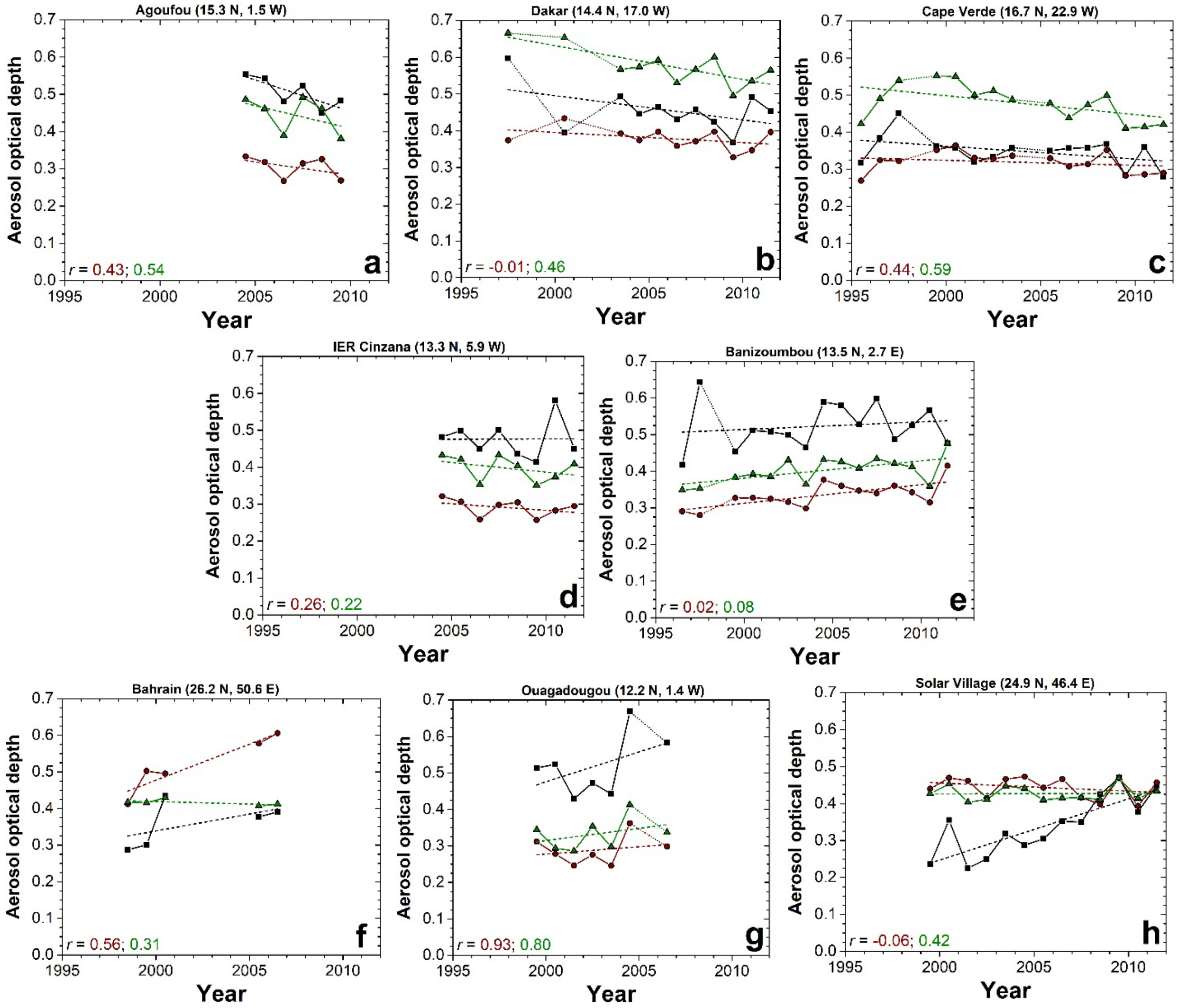

Supplementary Figure 1. Comparison of measured and modeled long-term AOD trends at AERONET stations for which these long-term changes are dominated by changes in dust aerosols. Shown are the annually-averaged aerosol optical depth (AOD) measured by AERONET (black squares and lines) and modeled by both the BASE-AERONET (brown circles and lines) and PHYS-AERONET (green triangles and lines) simulations. Dashed lines denote linear fits to the measured and modeled AOD, thus quantifying the long-term trends at each station. The eight panels are arranged in order of increasing measured long-term trend. The Pearson correlation coefficients (*r*) between the measured and modeled annually-averaged AOD are noted for the BASE-AERONET (green numbers) and PHYS-AERONET (brown numbers) simulations.

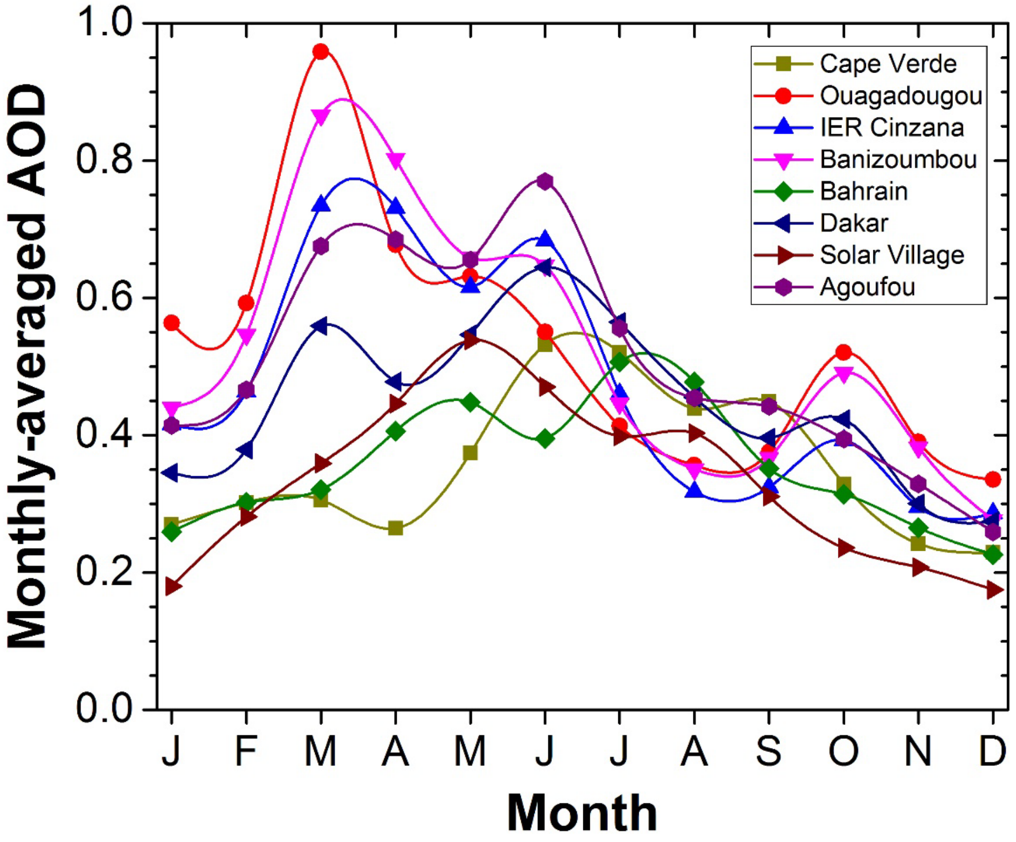

Supplementary Figure 2. The seasonal cycle at dusty AERONET stations. Shown are the seasonal cycles of measured monthly-averaged AOD at the eight AERONET sites that are both dust-dominated, and for which long-term changes in AOD are likely due to changes in dust aerosol (see Supplementary Methods).

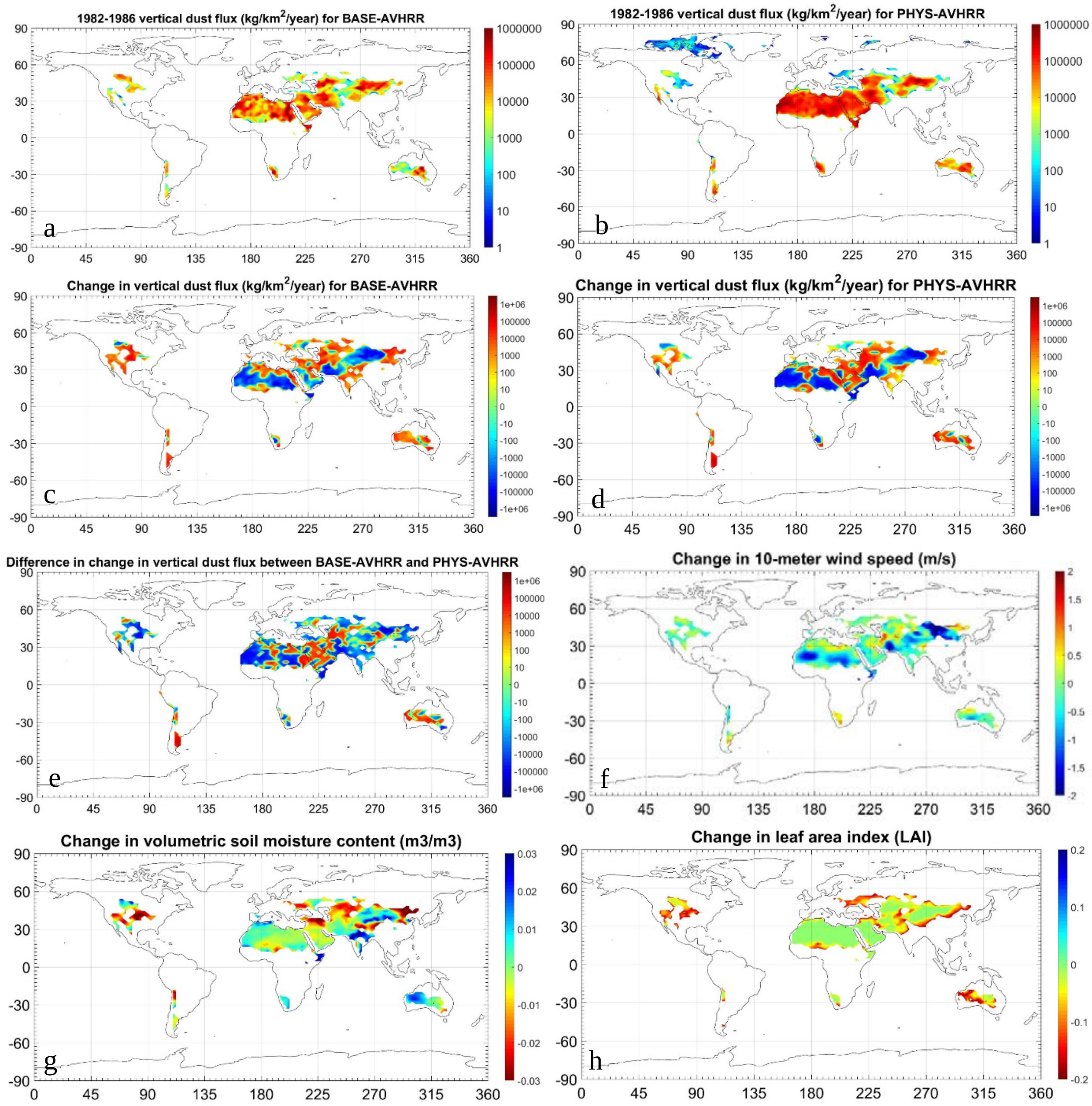

Supplementary Figure 3. Changes in dust flux, and its drivers, for the CESM BASE-AVHRR and PHYS-AVHRR simulations. Mean annual dust flux for the period 1982-1986 for (**a**) the BASE-AVHRR and (**b**) the PHYS-AVHRR simulations. Also shown are dust flux changes between the periods 1982 – 1986 and 2004 – 2008 for (**c**) the BASE-AVHRR and (**d**) the PHYS-AVHRR simulations, and (**e**) the difference in the dust flux change between the two simulations. Shown for comparison are also the change in (**f**) 10-meter wind speed, (**g**) volumetric soil moisture content, and (**h**) leaf area index (LAI). For panels (**c**), (**d**), and (**f**) – (**h**), warm (red) and cold (blue) colors denote changes that respectively produce increases and decreases in the vertical dust flux, and changes are shown only for regions that emitted dust in the year 2000. For panel (**e**), warm (red) colors denote areas for which the change in the PHYS-AVHRR simulations was larger (more positive) than for the BASE-AVHRR simulation.

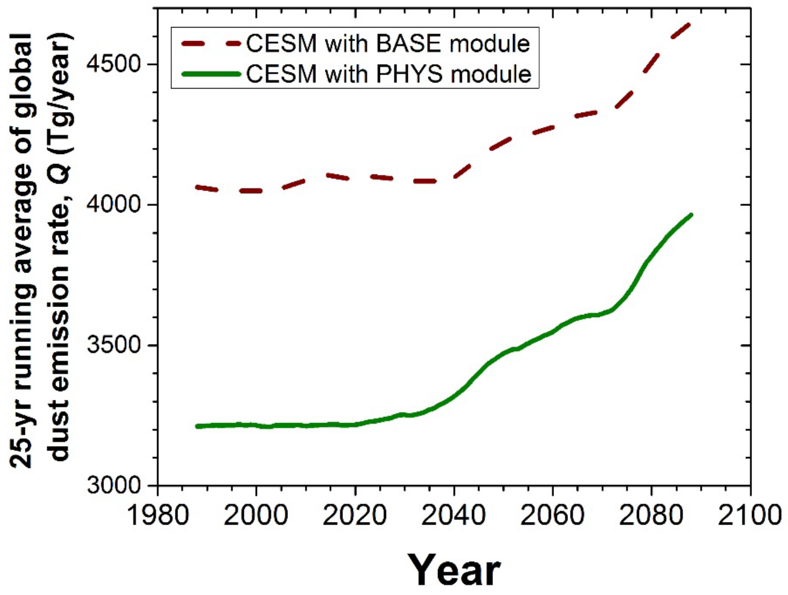

Supplementary Figure 4. Simulations of the response of the global dust emission rate to climate changes. The 25-year running average of the global dust emission rate, simulated using CESM/CLM with both the BASE (brown dashed line) and the PHYS (green solid line) dust emission modules. The two simulations have different present climate emission rates because for each run the global tuning parameter that scales the global dust emission was calibrated to eliminate the bias with AERONET AOD measurements in dusty regions, as described in Ref. [3].

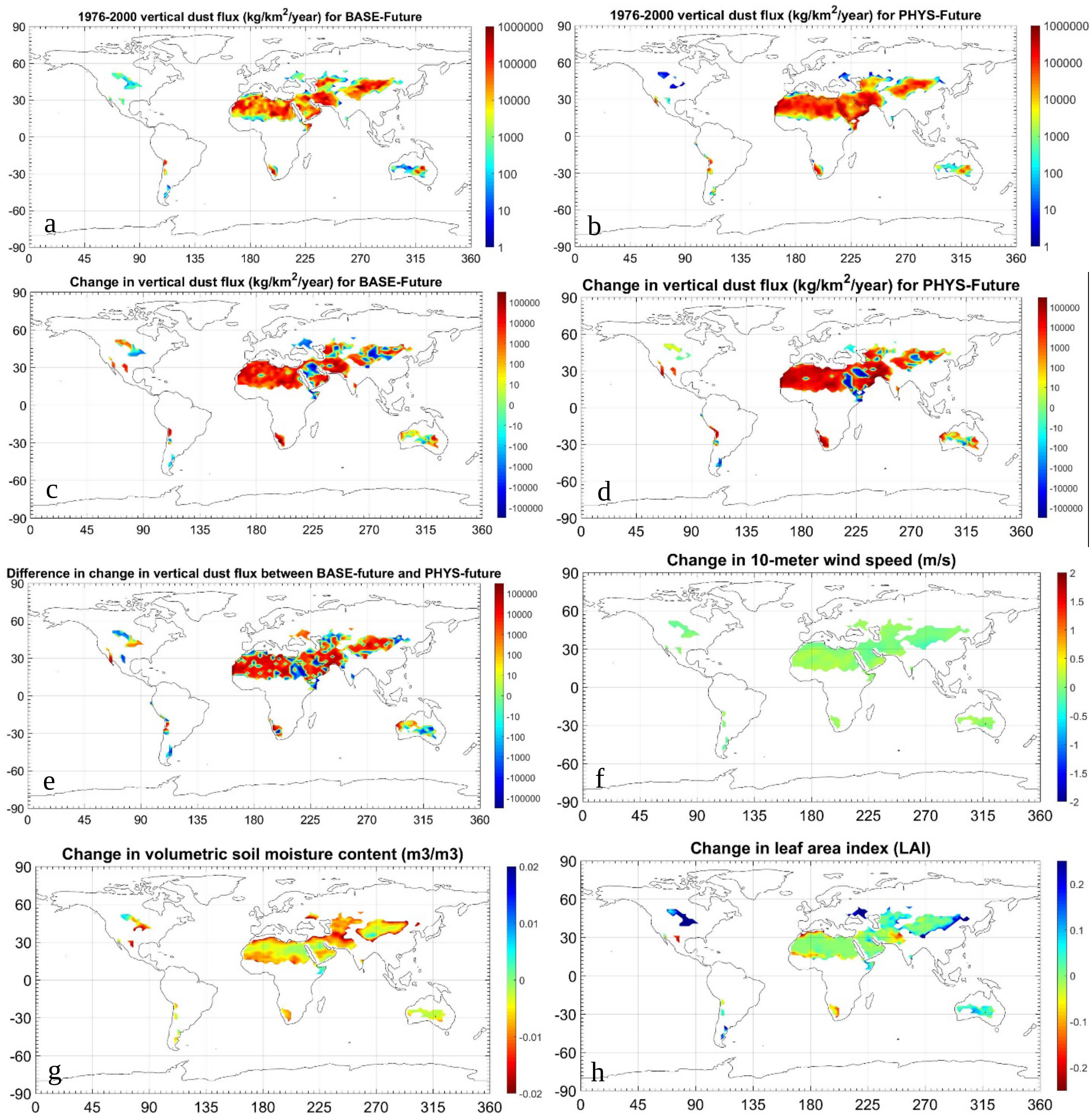

Supplementary Figure 5. Changes in dust flux, and its drivers, for the CESM BASE-Future and PHYS-Future simulations. Mean annual dust flux for the period 1976 – 2000 for (**a**) the BASE-Future and (**b**) the PHYS-Future simulations. Also shown are dust flux changes between the periods 1976 – 2000 and 2076 – 2100 in the dust flux for (**c**) the BASE-AVHRR and (**d**) the PHYS-AVHRR simulations, and (**e**) the difference in the dust flux change between the two simulations. Shown for comparison are also the change in (**f**) 10-meter wind speed, (**g**) volumetric soil moisture content, and (**h**) leaf area index (LAI). For panels (**c**), (**d**), and (**f**) – (**h**), warm (red) and cold (blue) colors denote changes that respectively produce increases and decreases in the vertical dust flux, and changes are shown only for regions that emitted dust in the period 2004-2008. For panel (**e**), warm (red) colors denote areas for which the change in the PHYS-Future simulation was larger (more positive) than for the BASE-Future simulation.

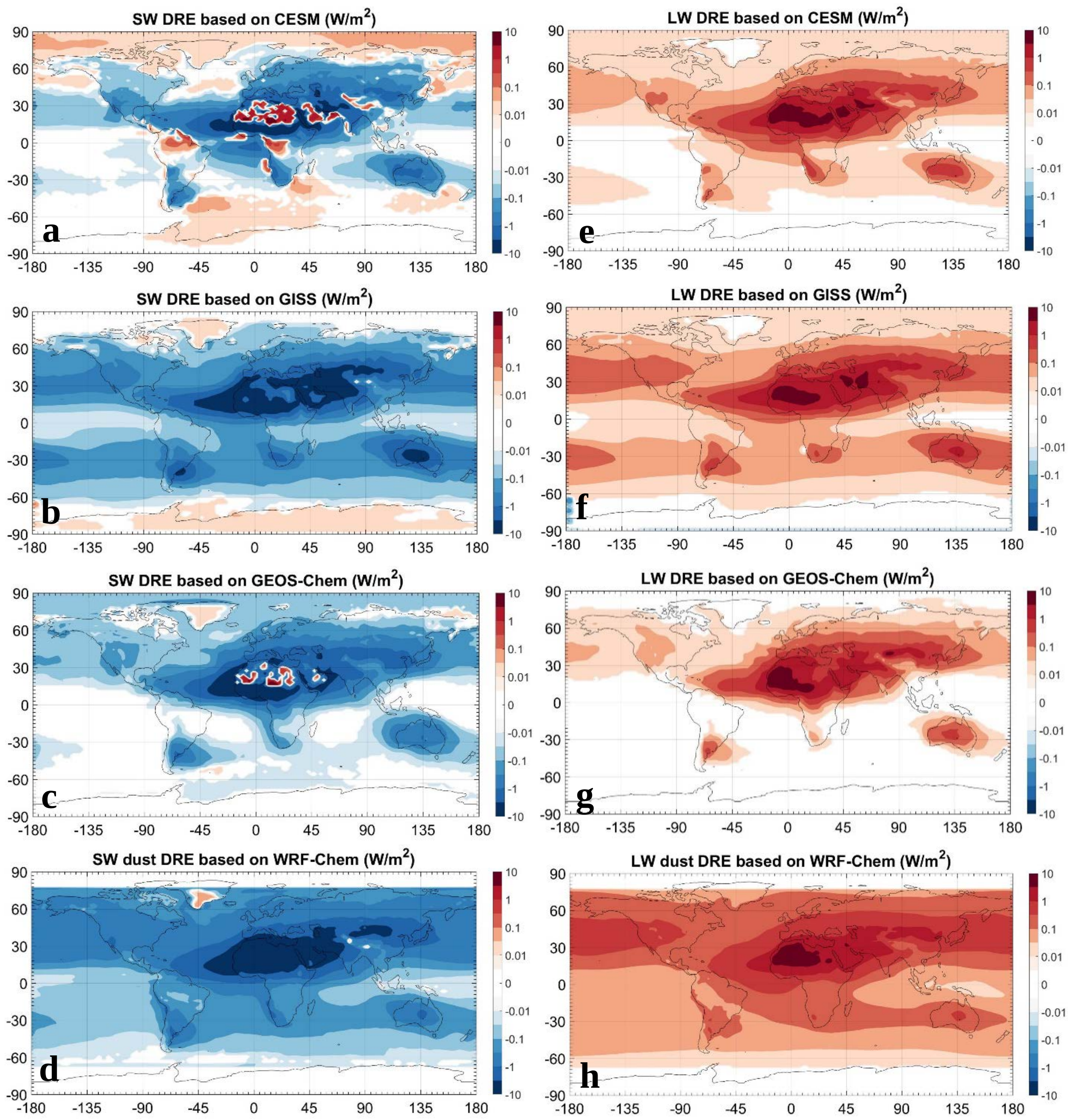

Supplementary Figure 6. The regional dust direct radiative effect (DRE), based on different climate model simulations. Shown are the present climate dust DRE in the SW (**a-d**) and LW (**e-h**), calculated by correcting different climate model simulations using constraints on the size-resolved dust optical depth (see Eqs. 7 and 8). Results are shown for simulations by the CESM (**a**, **e**), GISS (**b**, **f**), GEOS-Chem (**c**, **g**), and WRF-Chem (**d**, **h**) models[4].

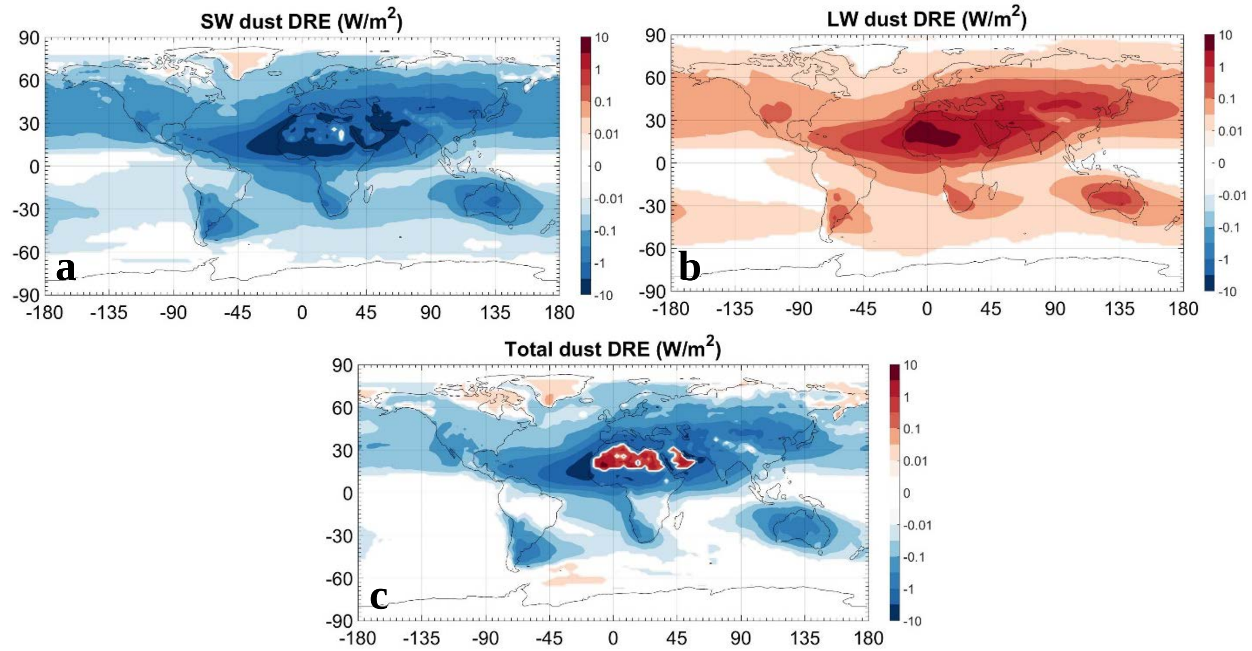

Supplementary Figure 7. The model-averaged regional dust direct radiative effect (DRE). Shown are the present climate dust DRE in (**a**) the SW ($\tilde{\zeta}_{0,\mathrm{SW}}$; see Eq. 10) and (**b**) LW spectra ($\tilde{\zeta}_{0,\mathrm{LW}}$; see Eq. 11), calculated from the average regional DRE derived from four different simulations (see Supplementary Figure 6). Also shown is the total DRE ($\tilde{\zeta}_{0,\mathrm{SW}} + \tilde{\zeta}_{0,\mathrm{LW}}$). Regional differences in DRE are largely driven by changes in surface albedo and dust loading.

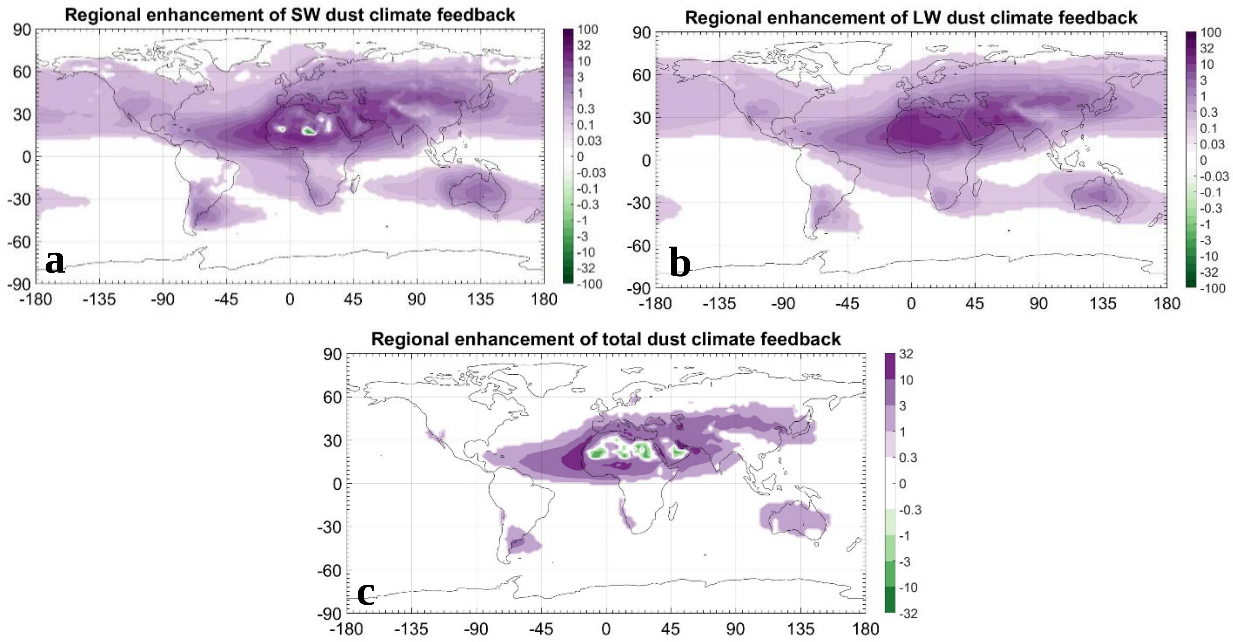

Supplementary Figure 8. **Enhancement of the regional over the globally-averaged direct dust-climate feedback feedback, $\tilde{E}_{\lambda}$.** Shown are the regional enhancements of the dust climate feedback (see Eq. 6) in (**a**) the SW spectrum ($\tilde{\zeta}_{0,\mathrm{SW}} / \zeta_{0,\mathrm{SW}}$), (**b**) the LW spectrum ($\tilde{\zeta}_{0,\mathrm{LW}} / \zeta_{0,\mathrm{LW}}$), and (**c**) the total feedback ($\tilde{\zeta}_{0} / \zeta_{0}$). As with the dust DRE, regional differences in the dust climate feedback enhancement are largely driven by changes in surface albedo and dust loading.

Supplementary References

1 Eck, T. F. *et al.* Wavelength dependence of the optical depth of biomass burning, urban, and desert dust aerosols. *Journal of Geophysical Research-Atmospheres* **104**, 31333-31349, doi:10.1029/1999jd900923 (1999).

2 Dubovik, O. *et al.* Variability of absorption and optical properties of key aerosol types observed in worldwide locations. *J. Atmos. Sci.* **59**, 590-608 (2002).

3 Kok, J. F., Albani, S., Mahowald, N. M. & Ward, D. S. An improved dust emission model - Part 2: Evaluation in the Community Earth System Model, with implications for the use of dust source functions. *Atmospheric Chemistry and Physics* **14**, 13043-13061, doi:10.5194/acp-14-13043-2014 (2014).

4 Kok, J. F., Ridley, D. A., Miller, R. L., Zhao, C. & Ward, D. S. (Zenodo, http://doi.org/10.5281/zenodo.268131, 2017).
